# The effect of ApoE ε4 on longitudinal brain region-specific glucose metabolism in patients with mild cognitive impairment: a FDG-PET study

**DOI:** 10.1016/j.nicl.2019.101795

**Published:** 2019-03-28

**Authors:** Manish D. Paranjpe, Xueqi Chen, Min Liu, Ishan Paranjpe, Jeffrey P. Leal, Rongfu Wang, Martin G. Pomper, Dean F. Wong, Tammie L.S. Benzinger, Yun Zhou, for the Alzheimer's Disease Neuroimaging Initiative

**Affiliations:** a The Russell H. Morgan Department of Radiology and Radiological Science, Johns Hopkins University School of Medicine, Baltimore, MD 21287, United States; b Department of Nuclear Medicine, Peking University First Hospital, Beijing, China; c Icahn School of Medicine at Mount Sinai, NY, New York, United States; d Mallinckrodt Institute of Radiology, Washington University in St. Louis School of Medicine, St. Louis, MO 63110, United States

**Keywords:** Alzheimer's disease, Mild cognitive impairment, ApoE ε4, FDG PET, Longitudinal, Partial volume correction, AD, Alzheimer's Disease, ADD, Alzheimer's Disease Dementia, ADNI, Alzheimer's Disease Neuroimaging Initiative, ApoE, Apolipoprotein E, CDR, Clinical Dementia Rating, GWAS, Genome-wide association study, FAQ, Functional Activities Questionnaire, FWER, Family-wise error rate, FWHM, Full width at half maximum, GDS, Geriatric Depression Scale, MCI, Mild Cognitive Impairment, MMSE, Mini-Mental State Exam, NPIQ, Neuropsychiatric Inventory Questionnaire, PVC, Partial Volume Correction, ROI, Region of interest, SUVR, Standardized uptake value ratio, SPM, Statistical parametric mapping

## Abstract

While the ApoE ε4 allele is a known risk factor for mild cognitive impairment (MCI) and Alzheimer's disease, brain region specific effects remain elusive. In this study, we investigate whether the ApoE ε4 allele exhibits brain region specific effects in longitudinal glucose uptake among patients with MCI from the Alzheimer's Disease Neuroimaging Initiative (ADNI). Preprocessed FDG PET images, MRIs, and demographic information were downloaded from the ADNI database. An iterative reblurred Van Cittertiteration method was used for partial volume correction (PVC) on all PET images. Structural MRIs were used for PET spatial normalization and region of interest (ROI) definition in standard space. Longitudinal changes in ROI FDG standardized uptake value ratio (SUVR) relative to cerebellum in 24 ApoE ε4 carriers and 24 age-matched ApoE ε4 non-carriers were measured for up to 84-months (median 72 months, SD = 11.2 months) and compared using a generalized linear mixed effects model controlling for gender, education, baseline age, and follow-up period. Additionally, voxelwise analysis was performed by implementing a paired *t*-test comparing matched baseline and 72 month FDG SUVR images in ApoE carriers and non-carriers separately. Results with PVC were compared with ones from non-PVC based analysis. After applying PVC, the superior fontal, parietal, lateral temporal, medial temporal, caudate, thalamus, and post-cingulate, and amygdala regions had greater longitudinal decreases in FDG uptake in ApoE ε4 carriers with MCI compared to non-carriers with MCI. Similar forebrain and limbic clusters were found through voxelwise analysis. Compared to the PVC based analysis, fewer significant ApoE-associated regions and clusters were found in the non-PVC based PET analysis. Our findings suggest that the ApoE ε4 genotype is associated with a longitudinal decline in glucose uptake in 8 forebrain and limbic brain regions in the context of MCI. In conclusion, this 84-months longitudinal FDG PET study demonstrates a novel ApoE ε4-associated brain-region specific glucose metabolism pattern in patients with MCI. Partial volume correction improved FDG PET quantification.

## Highlights

• Longest FDG-PET study in MCI to date (median follow-up of 72 months)
• Voxelwise partial volume correction improved longitudinal FDG-PET quantification
• Discovery of a novel ApoE ε4-associated hypometabolic pattern in MCI

## 1 Introduction

Alzheimer's disease (AD) is characterized by memory loss and cognitive decline (Jahn, 2013; Rajan et al., 2015; Small et al., 2000). Pathological signatures of AD include amyloid peptide plaques, neurofibrillary tangles, and synaptic dysfunction (Mosconi et al., 2010; Thambisetty et al., 2010a). Neuroimaging studies have established glucose hypometabolism and bilateral temporoparietal hypoperfusion in subjects who later develop AD (Lehtovirta et al., 1998; Mosconi et al., 2004).

Previous studies have shown the dose dependent role of apolipoprotein E (ApoE) ε4 allele as a risk factor for developing Alzheimer's disease, increasing the risk of AD by 2.6 to 14.9 fold and lowering the onset by 7 to 15 years (Farrer et al., 1997; Thambisetty et al., 2010a). By age 85, the lifetime risk estimate of developing Alzheimer's Disease Dementia (ADD) is 60–70% for ε4/ε4 homozygotes and 20–30% for ε4/ε3 heterozygotes (ApoE carriers) (Genin et al., 2011). In comparison, the lifetime risk for developing ADD by the age of 85 for ε3 / ε3 individuals (ApoE non-carriers) is only ~10%, suggesting that ApoE is moderately penetrant with a semi-dominant inheritance pattern (Genin et al., 2011). In the brain, ApoE is produced by non-neuronal cells, including astroglia and microglia, and functions by delivering cholesterol to neurons (Liu et al., 2013; Zhang et al., 2013). ApoE ε4, together with ApoE ε2 and ε3, comprise the three major allelic forms of ApoE each differing at amino acid positions 112 and 158. In addition to its known association with cognitive decline and Alzheimer's, including recent GWAS studies identifying ApoE ε4 as the main genetic determinant of Alzheimer's, polymorphic forms of ApoE ε4 have been linked to diverse physiological consequences including atherosclerosis, faster disease progression in multiple sclerosis, poor outcomes in traumatic brain injury, sleep apnea and a higher risk for type III hyperlipoproteinemia (Breslow et al., 1982; Elias-Sonnenschein et al., 2011; Feussner et al., 1998; Friedman et al., 1999; Kadotani et al., 2001; Schmidt et al., 2002). Despite biochemical, transgenic mouse and clinical studies offering potential explanations for the role of ApoE ε4 in AD progression, precise molecular mechanisms underlying ApoE ε4-mediated AD risk remains elusive.

Pathological changes in patients who develop AD often start decades before the first visible symptoms of AD. These changes can first present as mild cognitive impairment (MCI), a condition defined as cognitive decline above the age- and education-adjusted normal but below the threshold for ADD (Petersen et al., 2001, 1999; Winblad et al., 2004). Patients with MCI have an annual conversion rate to AD of 10–15%, in contrast to only 1–2% among healthy individuals (Bruscoli and Lovestone, 2004; Chen et al., 2017; Petersen et al., 2001, 1999). The presence of the ApoE ε4 allele increases the odds of converting from amnestic MCI to ADD by 4.1 fold (95% CI: 1.2–13.6) (Petersen et al., 2005; Scarabino et al., 2016). Yet in spite of these findings, little is known about how the presence of the ApoE ε4 allele induces pathological changes in the context of MCI. Given our existing knowledge of the exquisite region-specific patterns of brain atrophy, glucose hypometabolism, spread of tau and amyloid burden in AD progression, we asked whether the ApoE ε4 allele, as the largest genetic determinant of AD risk, also elicits similar brain-region specific changes in MCI which often precedes Alzheimer's (Chan et al., 2003; Cope et al., 2018; Drzezga et al., 2005; Grijalva-Eternod et al., 2012; Knopman et al., 2014; Mecca et al., 2018; Misra et al., 2009; Mosconi et al., 2009).

Functional neuroimaging is a valuable non-invasive tool to monitor disease progression and brain metabolic changes in various neurodegenerative disease, including AD, Parkinson's disease and HIV-associated neurocognitive disorders (Chen et al., 2016; Dickens et al., 2017; Gao et al., 2016; Niethammer et al., 2012). In particular, FDG-PET imaging allows for the characterization of ApoE ε4-mediated phenotypic changes in AD and MCI, including its association with glucose hypometabolism (Rosén et al., 2013). Cross sectional studies including work by Reiman et al. have used FDG imaging to establish the dose-dependent effect of ApoE ε4 on hypometabolism in non-demented adults (Reiman et al., 1996). Longitudinal studies such as work by Thambisetty et al. used oxygen-15 PET to compare changes in cerebral blood flow in 29 non-demented ApoE ε4 carriers and 65 non-carriers (Thambisetty et al., 2010a). They found greater longitudinal decline in cerebral blood flow in ApoE ε4 carriers compared to non-carriers in the temporal, parietal, and frontal cortices. While these studies are important in establishing the association of the ApoE ε4 allele with increased glucose hypometabolism, these studies were performed in the context of normal aging.

These existing studies, including work by Thambisetty and Reiman, have helped to show ApoE ε4 dose-dependent associations between cerebral blood flow decline and hypometabolism, both validated readouts of AD (Reiman et al., 1996; Thambisetty et al., 2010a). However, there exists a clear lack of >4 years longitudinal FDG studies, which are critical for studying the role of ApoE ε4 in increasing the risk of disease over time. In contrast to previous studies, a up to 84 month (median 72 month) follow up period was used in the present study with partial volume correction (PVC) to minimize inaccuracies in PET measurement due to changes in tissue volume or low spatial resolution. Partial volume effect is particularly a concern in small brain regions or in longitudinal studies in which atrophy can lead to an underestimation or overestimation of the true changes in quantitative PET (Jonasson et al., 2017). Several recent analyses have highlighted the importance of correcting for partial volume effects in longitudinal brain PET studies (Dukart and Bertolino, 2014; Fouquet et al., 2009). In the present study, we sought to characterize regional differences in glucose metabolism over time between ApoE ε4 carriers and non-carriers with MCI after PVC correction and compared these results with non-PVC analysis. Specifically, we investigated regional associations between the ApoE ε4 genotype and longitudinal changes in glucose uptake, measured using FDG PET, over a median 72-month follow-up period in 48 subjects with MCI (24 ApoE ε4 carriers and 24 non-carriers) from the Alzheimer's Disease Neuroimaging Initiative (ADNI). We identified a novel ApoE ε4–dependent longitudinal pattern of glucose hypometabolism in MCI patients.

## 2 Methods

### 2.1 Subjects

We initially queried the ADNI 1,2, and GO databases for individuals with at least a 48-month follow-up and FDG-PET and MRI data at each study visit. This query was performed for a previous study conducted in our group involving healthy controls and MCI individuals (Chen et al., 2016). From this cohort, we selected all available MCI individuals with FDG-PET and MRI at each scanning visit.

Consent was obtained from all study participants prior to the start of the study. The study was conducted with Institutional Review Board approval. We followed exclusion criteria determined by ADNI. Specifically, subjects with neurological disease other than Alzheimer's, including Parkinson's, multi-infarct dementia, Huntington's, normal pressure hydrocephalus, brain tumor, progressive supranuclear palsy, history of seizure, subdural hematoma, multiple sclerosis, history of head trauma, and known brain structural abnormalities, were excluded from our study. Additionally, subjects with evidence of infection, focal lesions, or infarction on MRI were excluded. Individuals with pacemakers, heart valve, or other foreign objects/implants in the body were excluded. Patients with history of major depression, bipolar disorder, schizophrenia, alcohol or substance abuse or those that are currently using psychoactive medications were excluded.

At baseline, all MCI subjects had a subjective memory concern reported by a clinician and abnormal memory function on the education-adjusted Logical Memory II subscale. All MCI subjects were required to have a clinical dementia rating of 0.5 or higher. Further, all MCI subjects were deemed to have cognitive and functional performance that was sufficiently intact to not merit a diagnosis of ADD by the site physician.

A complete list of ADNI exclusion/inclusion procedures can be found at https://adni.loni.usc.edu/wp-content/uploads/2008/07/adni2-procedures-manual.pdf

All data were downloaded from the ADNI clinical data repository (http://www.loni.ucla.edu/ADNI/). ADNI was launched in 2003 as a public-private consortium. Informed consent was obtained by all study participants at the time of enrollment for imaging data, genetic sample collection and clinical questionnaires. The primary goal of ADNI is to test whether serial MRI, PET, other biological markers, and clinical and neuropsychological assessments can be combined to measure the progression of MCI and early AD.

### 2.2 ApoE genotyping

Peripheral blood (10 mL) was collected from study participants to be used for ApoE ε4 genotyping. Restriction enzyme isoform genotyping was used on DNA extracts to test for the presence of the ApoE ε4 genotype, as described previously (Hixson and Vernier, 1990). ApoE ε4 carriers (*n* = 24) were defined as individuals with at least one ε4 allele (either ε4/ ε4, ε4/ ε3 or ε4/ ε2). Non-carriers (n = 24) were defined as individuals with no ε4 allele.

### 2.3 Neuropsychological testing

The Neuropsychiatric Inventory questionnaire (Kaufer et al., 2000), Global Clinical Dementia Rating (Hughes et al., 1982), Mini-Mental State Examination (Pangman et al., 2000), Functional Assessment Questionnaire (Pfeffer et al., 1982), Alzheimer's Disease Assessment Scale and Geriatric Depression Scale (Pangman et al., 2000) were administered during each scanning visit to assess cognitive decline that can accompany Alzheimer's disease.

### 2.4 MRI and PET acquisition and processing

T1-weighted MRI, and pre-processed FDG PET images were downloaded from http://adni.loni.usc.edu/. Detailed methods in FDG PET and MRI imaging and collection can be found at http://adni.loni.usc.edu/methods-/pet-analysis/pre-processing. Before downloading the images, the PET images were aligned, averaged, reoriented and then interpolated into a standard image and voxel size (image volume 160 × 160 × 96, 1.5 × 1.5 × 1.5 mm in x, y, z), and smoothed to a uniform resolution of 8 mm in full width at half maximum (FWHM) by ADNI (Drzezga et al., 2005).

The downloaded PET images were then further processed using Statistical Parametric Mapping software (SPM12, Wellcome Department of Imaging Neuroscience, London, United Kingdom) and MATLAB (The MathWorks Inc.). All preprocessed PET images after PVC were coregistered to structural MRI images acquired at each follow up. The MRI images were normalized to standard Montreal Neurologic Institute (MNI) space using SPM12 and the VBM8 toolbox with a MRI template (image volume: 121 × 145 × 121, voxel size: 1.5 × 1.5 × 15 mm in x, y, z). Transformation parameters determined by MRI spatial normalization were then applied to the coregistered PET images for PET spatial normalization. A total of 25 regions of interest (ROIs) including cerebellum gray matter for reference tissue were manually drawn on the MRI template using PMOD software (PMOD Technologies Ltd., Zürich, Switzerland) in standard MNI space. These tracer- and study-specific ROIs have been described in our previous studies (Chen et al., 2016; Gao et al., 2016; Gottesman et al., 2016; Resnick et al., 2010; Zhou et al., 2007). A global cortex was defined as a union of the orbital frontal, prefrontal, superior frontal, lateral temporal, parietal, posterior precuneus, occipital, anterior cingulate, and posterior cingulate. Standard uptake value ratio (SUVR) images relative to the cerebellum were produced. A summary of ROIs imposed over a MRI in standard space is presented in Supplementary Fig. 2. ROI SUVRs were obtained by applying ROIs to SUVR images in the MNI space.

PVC was applied to the preprocessed PET images to correct or minimize potential underestimation in PET measurements due to low image resolution, especially for small tissues such as the amygdala and caudate. In brief, an iterative reblurred Van Cittertiteration method was used for PVC on the mean images, where a 3-D Gaussian kernel of 8 mm FWHM was used for spatial smoothing function h, step length α = 1.5, and the iteration was stopped if relative percent change of PVC images <1% (Tohka and Reilhac, 2008).

For comparison purposes, the above image processing including MRI-PET coregistration and spatial normalization, and ROI SUVR calculation were also applied to the downloaded preprocessed FDG PET images directly without PVC.

### 2.5 PET statistical analysis

ROI-based longitudinal changes in glucose uptake in ApoE ε4 carriers and non-carriers were evaluated by fitting a linear mixed-effects model in R (R version: 3.1.1; www.r-project.org). Each subject's sex, educational level, baseline age, follow-up period, and ApoE ε4 carrier status by time interaction (ApoE:time) were included as fixed effects. Subject ID was included as a random effect. The generalized linear mixed model was fit for each ROI FDG SUVR and the ApoE:time term was evaluated to identify regions with significantly different FDG uptake over time between ApoE ε4 carriers and non-carriers. FDG at 0, 6, 12, 18, 24, 36, 48, 60, 72, 84 months was included for a median follow-up time of 72 months.

Considering the increased variance in voxelwise SUVR measurements compared to ROI-based SUVRs, we implemented a paired *t*-test to increase statistical power in our voxelwise analyses. Specifically, we implemented a paired t-test comparing matched 72 months scans to baseline scans using SPM12 (cluster level FWER <0.05; uncorrected voxel *p* < .001) separately for ApoE ε4 carriers and non-carriers (Thambisetty et al., 2010a, Thambisetty et al., 2010b). The paired t-test design (in which we could compare two timepoints) also helped avoid confounding related to inter-subject variation. 72 months and baseline scans were chosen to attain a follow-up period of 72 months and remain consistent with the median follow-up time of 72 months used for the ROI-based analysis. Cluster extents for ApoE ε4 carriers and non-carriers were 135 voxels and 85 voxels, respectively. Voxels were assigned anatomical regions and Brodmann areas using Talairach Client (v2.4.3; talairach.org).

### 2.6 Data availability

All datasets used during the current study are available in the ADNI repository, adni.loni.usc.edu. Refer to Supplementary Table 1 for a list of ADNI subjects used in this study.

## 3 Results

### 3.1 Participant characteristics and longitudinal changes in neuropsychological performance

Following our selection criteria, we ended up with 24 ApoE ε4 carriers and 24 ApoE ε4 non-carriers with mild cognitive impairment (MCI) ranging from 55 to 85 years of age. All subjects underwent FDG PET scanning, structural MRI, and neuropsychological testing for a median follow-up period of 72 months (mean: 71.3 months, standard deviation (SD): 11.2 months; range: 48–84 months). The mean number of scans per participant was 7.5 (SD: 0.7 scans, range: 6–9 scans). The average time interval between follow-up scans was 10.9 months (SD = 1.3 months). Table S1 summarizes which scans were available for each participant. Statistical analysis showed no significant differences between the ApoE ε4 carriers and non-carriers in age, sex distribution, and education level at baseline (p: 0.12–0.90) (Table 1). No statistically significant differences were detected between carriers and non-carriers at baseline in any neuropsychological tests (p: 0.18–0.99).

**Table 1:** Demographic and statistics of neuropsychological performance at baseline for APOE ε4 carriers and non-carriers.

	**Baseline participant characteristics (*n*** **=** **48)**		
	**ApoE ε4 carrier (*n*** **=** **24)**	**ApoE ε4 non-carrier (n** **=** **24)**	***P* value**^a^
	Mean (Min, Max, SD)	Mean (Min, Max, SD)	
Number of ε4ε4 / ε4ε3/ ε4ε2 in ApoE ε4 carriers	6/16/02		
Number of ε3ε3 / ε3ε2/ in ApoE ε4 non-carriers	24/0/0		
Male / Female	18/6	16/8	0.53
Age	71.9 (55.3, 84,1.5)	75.6 (61.6, 85.4, 1.4)	0.12
Education years	15.6 (8, 20, 0.6)	15.1 (12, 20,0.6)	0.81
ADAS-cog TOTAL11^b^	9.9 (5.3, 20.3 0.8)	9.4 (5, 18, 0.7)	0.62
ADAS-cog TOTALMOD^b^	16.6 (8.3, 28.3, 1.1)	14.9 (9, 29, 1.4)	0.36
MMSE^c^	27.9 (24, 30, 0.3)	27.5 (24, 30, 0.3)	0.43
Global CDR^d^	0.5 (0.5, 0.5, 0)	0.5 (0.5, 0.5 0)	0.33
NPIQ Total Score^e^	2.4 (0, 17, 0.7)	1.5 (0, 6, 0.3)	0.3
FAQ Total Score^f^	3.0 (0, 9, 0.6)	2.6 (0, 13, 0.7)	0.69
GDS Total Score^g^	1.3 (0, 5, 0.3)	1.6 (0, 5, 0.3)	0.35

a *P* value represents statistical significance level of difference in ApoE ε4 carriers vs non-carriers.

b ADAS-Cog TOTAL 11 contains eleven items on the Alzheimers's Diseaes Assessment Scale including word recall, recognition, naming, etc. (range 0–70) and ADAS-Cog Total Mod includes all the eleven items plus delayed word recall and number cancellation (range 0–85).

c MMSE refers to the Mini-Mental State Examination.

d Global CDR refers to the Global Clinical Dementia Rating.

e NPIQ refers to the Neuropsychiatric Inventory questionnaire.

f FAQ refers to Functional Assessment Questionnaire.

g GDS refers to the Geriatric Depression Scale.

ApoE ε4 carriers had significantly greater cognitive decline compared to non-carriers over the follow-up period. Specifically, we fit a linear mixed effects model with cognitive test score for each subject at each timepoint as an outcome. Age at baseline, years of education, sex and ApoE:time interaction were included as covariates. We tested the effect of ApoE ε4 allele on cognitive decline in our study cohort by assessing the significance of ApoE:time interaction term and found that ApoE ε4 carriers exhibit significantly greater declines in MMSE, FAQ, CDR, GDS, and ADAS-cog11 and ADAS-cog13 compared to ApoE ε4 non-carriers over the study period (*p* < .05). We found no significant difference in longitudinal decline in NPIQ score between carriers and non-carriers.

We also compared the diagnosis status (ADD or MCI) of the study participants at baseline and last follow-up to see if there were differences in the proportion of ApoE carriers and non-carriers that converted to ADD. Among the ApoE ε4 carriers included in the study, at the last follow-up, 13 of 24 ApoE ε4 carriers had converted to ADD. There were also 24 APOE ε4 non-carriers scanned at baseline; at the last follow-up 9 of these 24 ApoE ε4 non-carriers had converted to ADD. The difference in the rates of conversion to ADD between ApoE ε4 carriers and ApoE ε4 non-carriers were near-significant (*p* = .123; one-tailed chi-square test).

**Supplementary Fig. 1:**
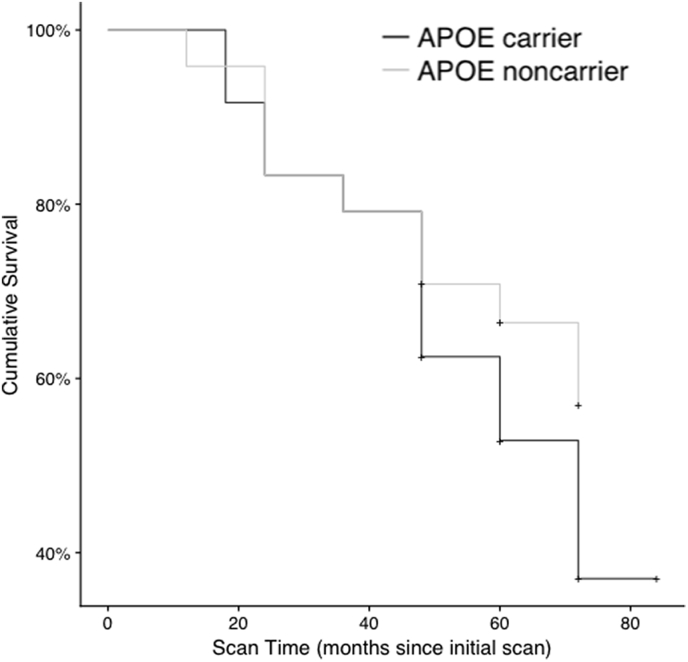
Survival plots of AD Progression in APOE Genotype Groups. Survival plots representing the percent of individuals with MCI were generated using a Kaplan Meier estimate. The survival plots represent 1- probability of converting to AD. The pluses indicate that at least one subject was censored during this interval. The differences in the survival of APOE ε4 carriers and non-carriers was not significant (*p* = .17; see Results) yet trended towards the APOE ε4 carriers having a reduced survival over time compared to non-carriers. We hypothesize that in light of existing research showing biomarkers, such as FDG, responding years before cognitive symptoms associated with AD, the participants in our study may have been observed at earlier stages in which changes in diagnosis status from MCI to AD were not significantly different between carriers and non-carriers (Jack et al., 2013).

We then potted a Kaplan-Meier survival curve (Supplementary Fig. 1) showing the conversion over time in ApoE ε4 carriers and noncarriers. Survival was defined as not converting to ADD. Individuals that left the study or died before the last follow up were marked as censored. The survival curve shows a cumulative survival of 37% (CI: 21% - 65%) for ApoE ε4 carriers and 57% (CI: 40% - 81%) for non-carriers at the end of the follow-up period. This analysis indicates a non-significant trend (*p* = .17; Fleming-Harrington test) towards the APOE carriers having an increased progression to ADD within the follow up period, consistent with existing studies (Moreno-Grau et al., 2017; Mosconi et al., 2004).

### 3.2 Longitudinal ROI-based FDG SUVR

**Fig. 1:**
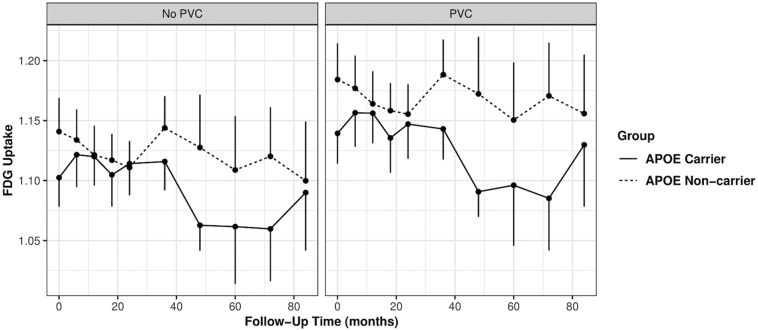
ROI-based analysis: Superior frontal cortex FDG SUVR dynamics in ApoE ε4 Carriers and Noncarriers with Mild Cognitive Impairment. Graphical representation of longitudinal FDG SUVR in ApoE ε4 carriers and non-carriers for PVC and non-PVC PET. FDG SUVR in the superior frontal cortex is plotted over time to show differences between ApoE ε4 carriers and non-carriers over the follow-up period. Differences in rates of FDG decline between ApoE ε4 carriers and non-carriers were assessed by fitting a linear mixed effects model to the ROI SUVRs, controlling for baseline age, education, follow-up interval and sex. (Table 2). The graph visually demonstrates an interaction effect between ApoE ε4 carrier status and follow-up time; ApoE ε4 carriers exhibit greater longitudinal decreases in superior frontal FDG compared to non-carriers. Due to partial volume effects, the contrast of longitudinal SUVRs between ApoE ε4 carriers and noncarriers was reduced in non-PVC PET data. Note, 96 months data is not shown because there were no ApoE ε4 carriers scans at this timepoint.

Longitudinal ROI FDG SUVR in ApoE ε4 carriers vs non-carriers was evaluated over a longitudinal follow-up (median = 72 months) interval using a generalized linear mixed effects model controlling for educational level, time of scan, baseline age, and gender. We found ROIs of the superior frontal cortex, amygdala, caudate, parietal cortex, posterior cingulate, lateral temporal and medial temporal and thalamic brain regions had significantly greater longitudinal decreases in FDG uptake in ApoE ε4 carriers compared to non-carriers for PVC based SUVRs (ApoE:time coefficient < 0; p < .05; Table 2). The superior frontal and amygdala failed to reach significance for non-PVC based SUVRs. The partial volume effects on the longitudinal FDG PET SUVR measurements are demonstrated in Fig. 1 using the superior frontal ROI. It clearly shows that the PVC increased contrast in longitudinal FDG ROI SUVR between ApoE ε4 carriers and non-carriers. Fig. 1 also shows an interaction effect between ApoE ε4 carrier status and follow-up time and confirms the results of the generalized linear mixed-effects model.

**Table 2:** ROI-based Analysis: Regions with Greater Decrease in ApoE ε4 Carriers vs. Non-Carriers in 72-month Longitudinal FDG PET Study Among Patients with MCI.

		**PVC**				**No PVC**				
	*P*^a^	Mean Baseline Scan (SEM)		Mean Percent Change Over Follow-Up Interval (SEM)^b^		*P*^a^	Mean Baseline Scan (SEM)		Mean Percent Change Over Follow-Up Interval (SEM)^b^	
Region		Carrier	Non- carrier	Carrier	Non- carrier		Carrier	Non- carrier	Carrier	Non- carrier
Superior Frontal	0.03	1.139	1.184	−0.58	−0.132	0.065	1.102	1.141	−0.503	−0.173
		0.025	0.03	0.284	0.305		0.024	0.028	0.275	0.289
Lateral Temporal	<0.001	0.971	1.009	−1.044	−0.363	0.001	0.937	0.975	−0.989	−0.413
		0.017	0.018	0.355	0.267		0.016	0.017	0.316	0.262
Medial Temporal	0.02	0.755	0.78	−0.824	−0.22	0.045	0.767	0.795	−0.929	−0.405
		0.017	0.016	0.335	0.421		0.017	0.017	0.335	0.396
Parietal	<0.001	1.034	1.054	−1.5	−0.31	<0.001	1.017	1.045	−1.353	−0.332
		0.019	0.024	0.353	0.344		0.019	0.024	0.328	0.326
Posterior Cingulate	0.002	1.231	1.287	−1.266	−0.632	0.037	1.28	1.346	−1.036	−0.642
		0.029	0.031	0.304	0.263		0.03	0.032	0.288	0.259
Amygdala	0.02	0.726	0.751	−0.428	0.291	0.094	0.77	0.806	−0.575	−0.121
		0.019	0.015	0.337	0.354		0.017	0.016	0.325	0.315
Caudate	<0.001	1.104	1.072	−1.686	−0.055	<0.001	0.947	0.935	−1.308	−0.167
		0.044	0.051	0.584	0.709		0.034	0.038	0.479	0.561
Thalamus	0.002	1.363	1.344	−0.803	1.152	0.003	1.196	1.19	−0.637	0.651
		0.027	0.067	0.665	0.582		0.026	0.049	0.481	0.525

a *P* value represents statistical significance level of difference in APOE carriers versus non-carriers adjusted for sex, education level, baseline age using a general linear mixed effects model.

b Percent decrease was averaged over all follow-up intervals for all participants to determine a mean percent decrease in FDG.

### 3.3 FDG SUVR image SPM analysis

**Fig. 2:**
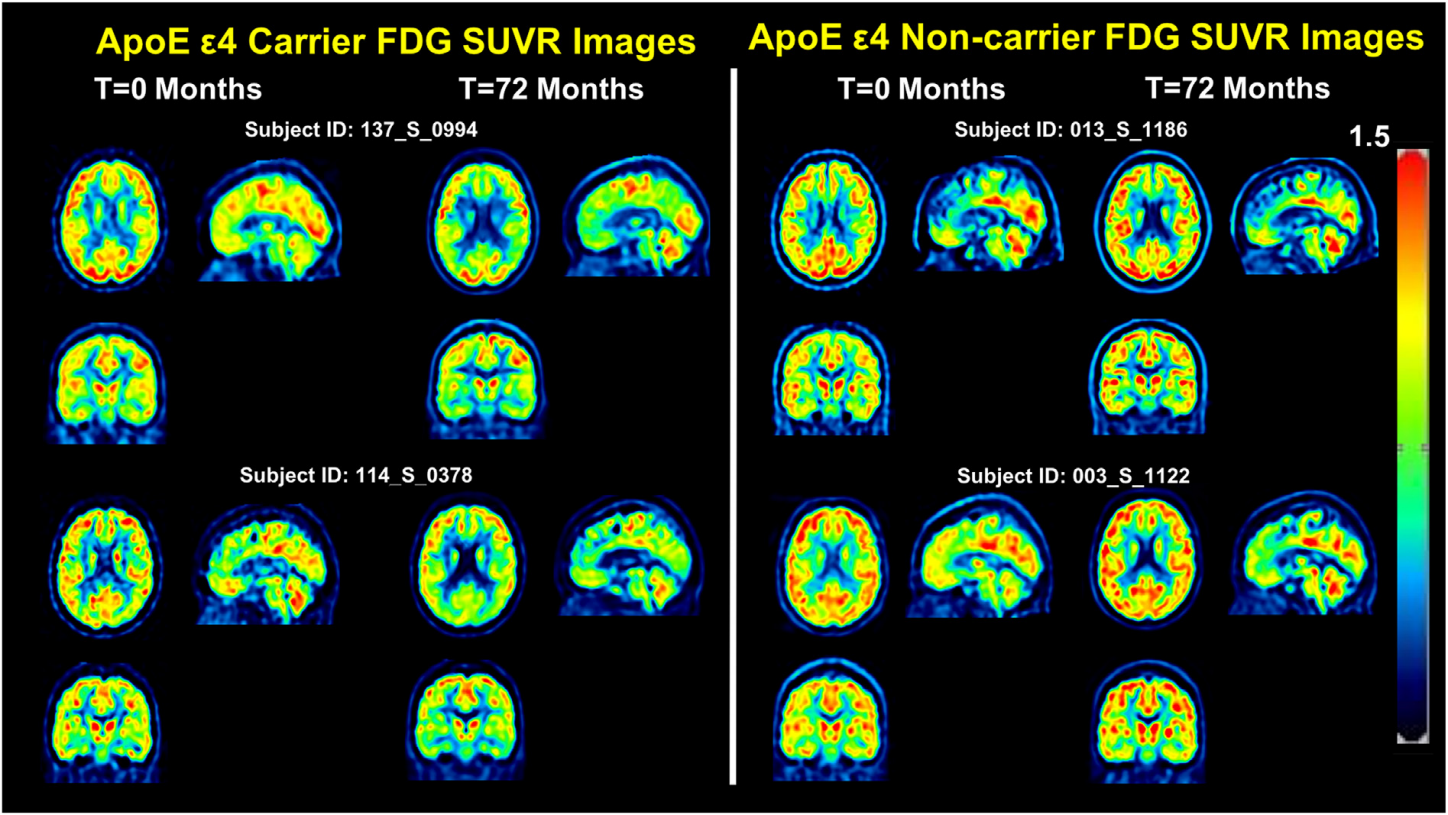
Representative single-subject SUVR images with PVC at baseline and 72 months**.** PVC-based FDG SUVR images at baseline and 72-month scans from two ApoE ε4 carriers and two non-carriers are displayed in MNI standard-space. The scans demonstrate typical longitudinal changes in FDG signal over the study period and confirm the results pooled voxel-wise results (Fig. 3).

**Fig. 3:**
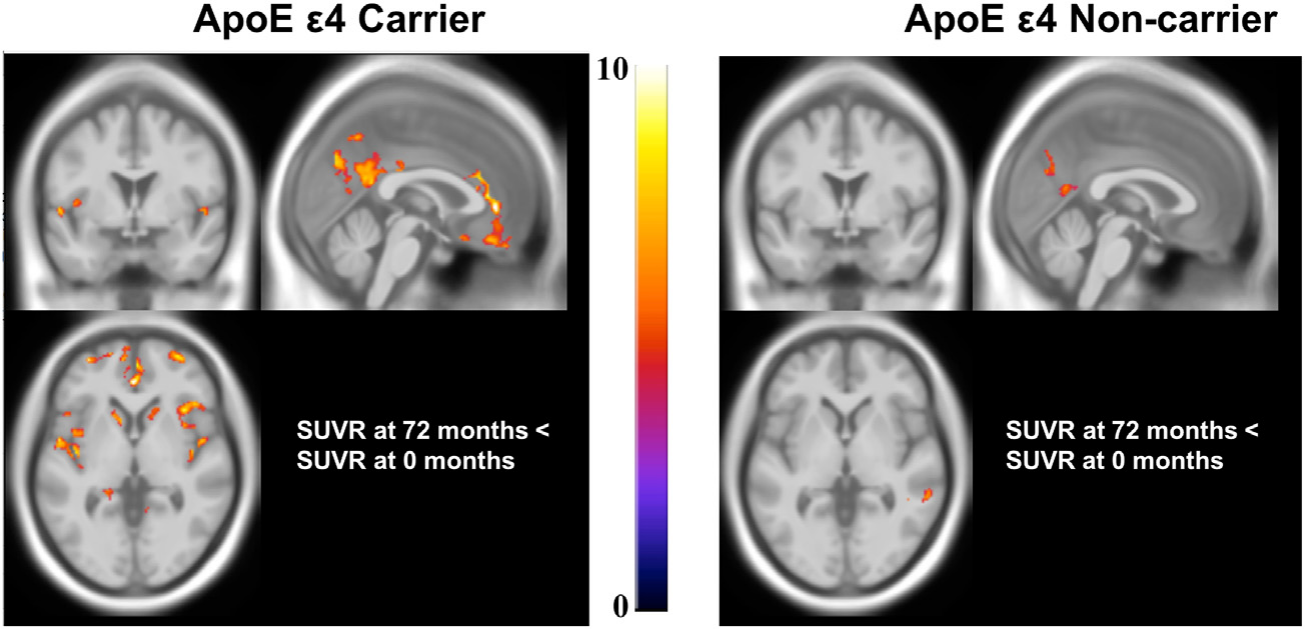
Voxelwise statistical analysis of PVC based FDG SUVR longitudinal changes in ApoE ε4 carriers and non-carriers. MNI152 standard-space T1-weighted average structural template overlay showing voxels with significant decrease in FDG at 72 months compared to baseline (cluster-level correction: FWER <0.05; voxel uncorrected *p* < .001). Overlay figures were generating by performing a paired *t*-test comparing 72 month PVC scans with matched baseline PVC scans in SPM separately for ApoE ε4 carriers (left) and non-carriers (right).

Voxel-wise FDG SUVR in ApoE ε4 carriers and non-carriers was evaluated separately using a paired *t*-test to compare 72 month scans with 0 month (baseline) scans. In ApoE ε4 carriers after PVC, 23 clusters corresponding to voxels in the frontal, temporal gyrus, precuneus, and anterior cingulate, posterior cingulate, caudate, thalamus, insula, and parahippocampal gyrus (cluster-level FWER p < .05; peak-level unadjusted p < .001; Table 3, Fig. 2, Fig. 3) exhibit significantly decreased FDG uptake at 72 months compared to 0 months. In contrast, in ApoE ε4 noncarriers after PVC, 4 clusters in the medial temporal, inferior temporal cortex, precuneus and posterior cingulate exhibited significantly decreased FDG uptake at 72 months compared to 0 months (cluster-level FWER p < .05; peak-level unadjusted p < .001; Table 3, Fig. 2, Fig. 3). These results are broadly replicated at the individual level using two representative ApoE ε4 carriers and two ApoE ε4 non-carriers in Fig. 2. It clearly demonstrates a hypometabolic pattern in the parietal, temporal, frontal, thalamus regions in the two ApoE ε4 carriers. Meanwhile, we observe minimal hypometabolic change in the two ApoE ε4 non-carriers. Voxel wise statistical results showing voxels with significant longitudinal decline in ApoE ε4 carriers and non-carriers are shown in Fig. 3. These voxelwise results indicate that ApoE ε4 carriers exhibit greater longitudinal decreases in FDG uptake in the frontal and parietal cortices, cingulate, striatal, limbic regions and support the results of our ROI-based linear mixed-effects analysis in which we considered FDG at 0, 6, 12, 18, 24, 36, 48, 60, 72, 84 months.

**Table 3:** Voxelwise analysis: Voxels with Significant Decrease at 72 months compared to Baseline Among ApoE ε4 carriers with MCI.

**Cluster level**			**Peak level**						
					MNI Coordinates^b^				
Corrected P value^a^	Cluster size (No. of Voxels)	Z	P value	Cohen's D	x	y	z	Region^c^	Brodmann Area
***ApoE ε4 Carrier SUVR at 72 month < SUVR at 0 month***									
0	196	5.33	<0.001	1.09	−24	−70	36	Precuneus	31
		3.17	0.001	0.65	−26	−82	34	Cuneus	19
0	303	5.14	<0.001	1.05	32	−73	44	Superior Parietal	7
		4.46	<0.001	0.91	32	−78	36	Precuneus	39
		4.12	<0.001	0.84	28	−67	38	Precuneus	7
0	1710	5.11	<0.001	1.04	0	41	0	Anterior Cingulate	32
		4.93	<0.001	1.01	3	24	19	Anterior Cingulate	24
		4.54	<0.001	0.93	10	51	12	Medial Frontal	10
0	730	4.97	<0.001	1.01	−40	−9	−3	Insula	13
		4.54	<0.001	0.93	−38	−9	−14	Angular gyrus	39
		4.51	<0.001	0.92	−57	−30	13	Superior Temporal	22
0.02	98	4.89	<0.001	1.00	−54	−61	−15	Fusiform	37
0	416	4.74	<0.001	0.97	51	6	−5	Superior Temporal	22
		4.51	<0.001	0.92	45	−10	7	Insula	13
		4.36	<0.001	0.89	39	−16	7	Insula	13
0	176	4.7	<0.001	0.96	−60	−39	21	Superior Temporal	22
		4.35	<0.001	0.89	−51	−36	16	Superior Temporal	22
0	218	4.69	<0.001	0.96	−32	56	1	Middle Frontal	10
		4.35	<0.001	0.89	−16	60	1	Superior Frontal	10
		3.95	<0.001	0.81	−24	53	9	Middle Frontal	10
0	2357	4.66	<0.001	0.95	6	−33	28	Cingulate	23
		4.55	<0.001	0.93	−9	−70	36	Precuneus	7
		4.52	<0.001	0.92	−3	−55	28	Cingulate	31
0	456	4.6	<0.001	0.94	−42	3	4	Insula	13
		4.49	<0.001	0.92	−39	15	−9	Inferior Frontal	47
		4.03	<0.001	0.82	−36	23	−8	Inferior Frontal	47
0.003	138	4.51	<0.001	0.92	−21	−40	−3	Parahippocampal	19
		4.02	<0.001	0.82	−33	−40	−11	Fusiform	36
0	529	4.46	<0.001	0.91	36	20	1	Insula	13
		4.16	<0.001	0.85	44	24	0	Inferior Frontal	47
		3.9	<0.001	0.80	36	9	0	Insula	13
0	316	4.46	<0.001	0.91	32	59	−2	Superior Frontal	10
		4.4	<0.001	0.90	22	63	6	Middle Frontal	10
		3.85	<0.001	0.79	28	60	10	Middle Frontal	10
0	338	4.38	<0.001	0.89	60	−19	13	Transverse Temporal	42
		4.13	<0.001	0.84	48	−25	12	Transverse Temporal	42
		3.66	<0.001	0.75	57	−13	9	Superior Temporal	22
0	230	4.37	<0.001	0.89	−51	−55	42	Inferior Parietal	40
		4.21	<0.001	0.86	−48	−63	40	Inferior Parietal	40
		3.8	<0.001	0.78	−44	−70	39	Precuneus	39
0.033	89	4.3	<0.001	0.88	2	−58	49	Precuneus	7
0.042	85	4.28	<0.001	0.87	51	11	22	Inferior Frontal	44
0.001	167	4.14	<0.001	0.85	27	50	13	Middle Frontal	10
		4.1	<0.001	0.84	28	50	24	Superior Frontal	10
0.001	158	4.1	<0.001	0.84	−12	17	−2	Caudate	48
		3.59	<0.001	0.73	−14	9	10	Caudate	48
0.001	149	4.06	<0.001	0.83	−8	56	0	Medial Frontal	10
		4.03	<0.001	0.82	−8	62	−15	Medial Frontal	11
		3.84	<0.001	0.78	−4	65	−5	Medial Frontal	10
0.024	95	3.96	<0.001	0.81	−60	−13	−32	Inferior Temporal	20
		3.43	<0.001	0.70	−51	−9	−29	Inferior Temporal	20
		3.39	<0.001	0.69	−57	−7	−36	Inferior Temporal	20
0.033	89	3.81	<0.001	0.78	−33	−49	40	Angular gyrus	39
0.03	91	3.59	<0.001	0.73	12	15	−2	Caudate	48
		3.53	<0.001	0.72	16	21	1	Caudate	48
		3.43	<0.001	0.70	10	11	6	Caudate	48

***ApoE ε4 Non-carrier SUVR at 72 month < SUVR at 0 month***									
0	312	4.35	<0.001	0.89	54	−39	−11	Medial temporal	21
		4.11	<0.001	0.84	52	−48	6	Medial temporal	37
		4.01	<0.001	0.82	60	−37	−3	Medial temporal	21
0.003	174	3.96	<0.001	0.81	−2	−72	28	Precuneus	31
		3.78	<0.001	0.77	−6	−72	21	Cuneus	18
		3.45	<0.001	0.70	0	−72	37	Precuneus	31
0.006	157	3.9	<0.001	0.80	58	−19	−18	Inferior Temporal Gyrus	21
		3.76	<0.001	0.77	57	−12	−21	Inferior Temporal Gyrus	21
		3.72	<0.001	0.76	58	−12	−11	Middle Temporal Gyrus	21
0.014	135	3.77	<0.001	0.77	−3	−46	7	Posterior Cingulate	30
		3.75	<0.001	0.77	0	−60	15	Posterior Cingulate	31

Results of the paired t-test comparing 72 month and matched baseline FDG scans in SPM are presented in Table 3. Among ApoE ε4 carriers, we found 23 clusters (cluster level FWER <0.05; peak level uncorrected p < .001) in which FDG at 72 months <0 months. Among ApoE ε4 non-carriers, we found 4 significant clusters after cluster-level correction.

a Cluster p value was corrected for FWER using SPM12.

b Coordinates are in MNI space based on the output from SPM12.

c MNI coordinates were mapped to the nearest gray matter brain region and Brodmann area using Talairach Client (v2.4.3; talairach.org).

**Fig. 4:**
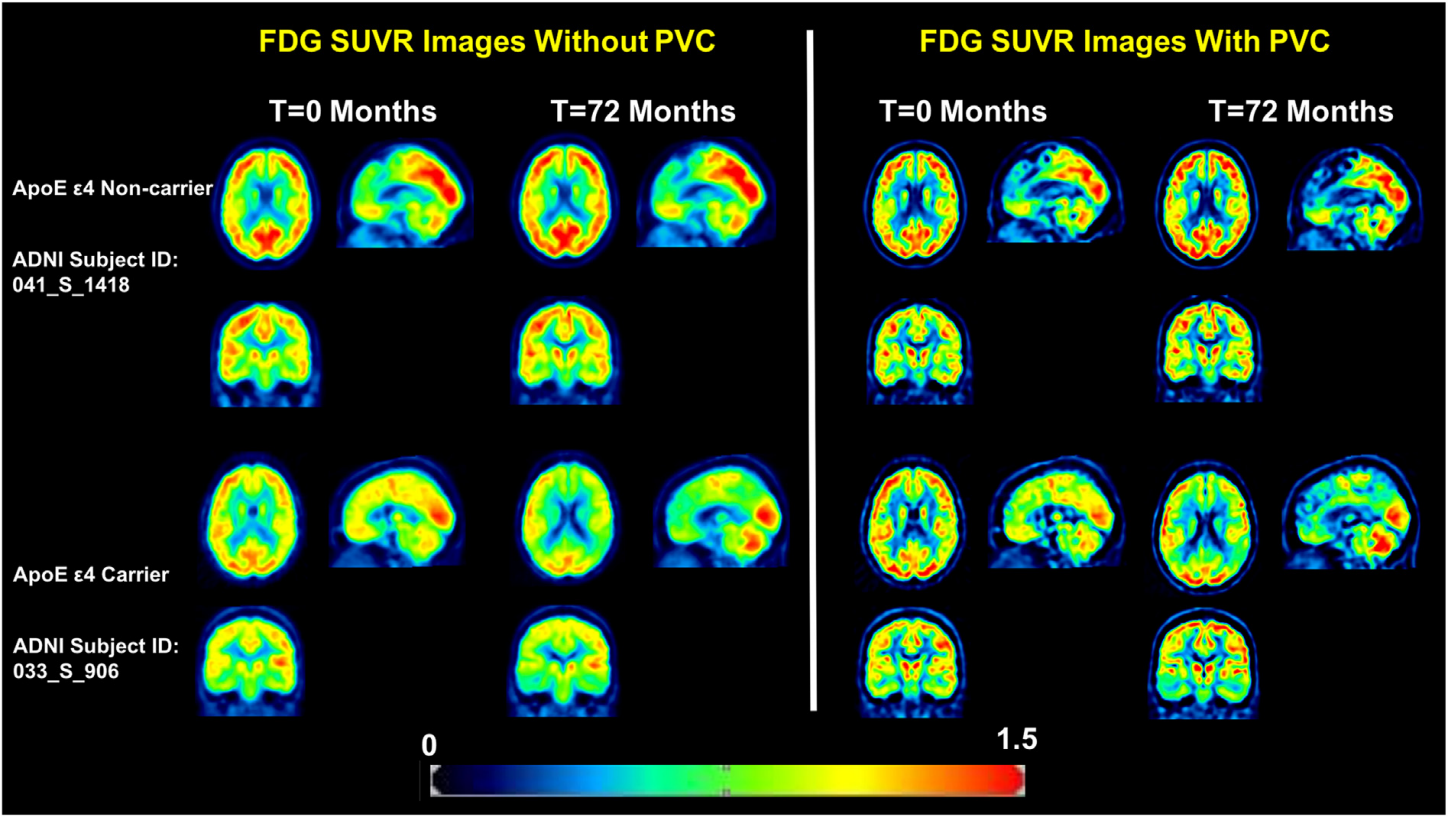
Typical single-subject FDG-PET Image with PVC and non-PVC. Representative cross-sectional slices from selected ApoE ε4 carriers (subject ID: 041_S_1418) and non-carriers (subject ID: 033_S_0906) with and without PVC are displayed to demonstrate the effect of PVC at the single-subject level.

**Fig. 5:**
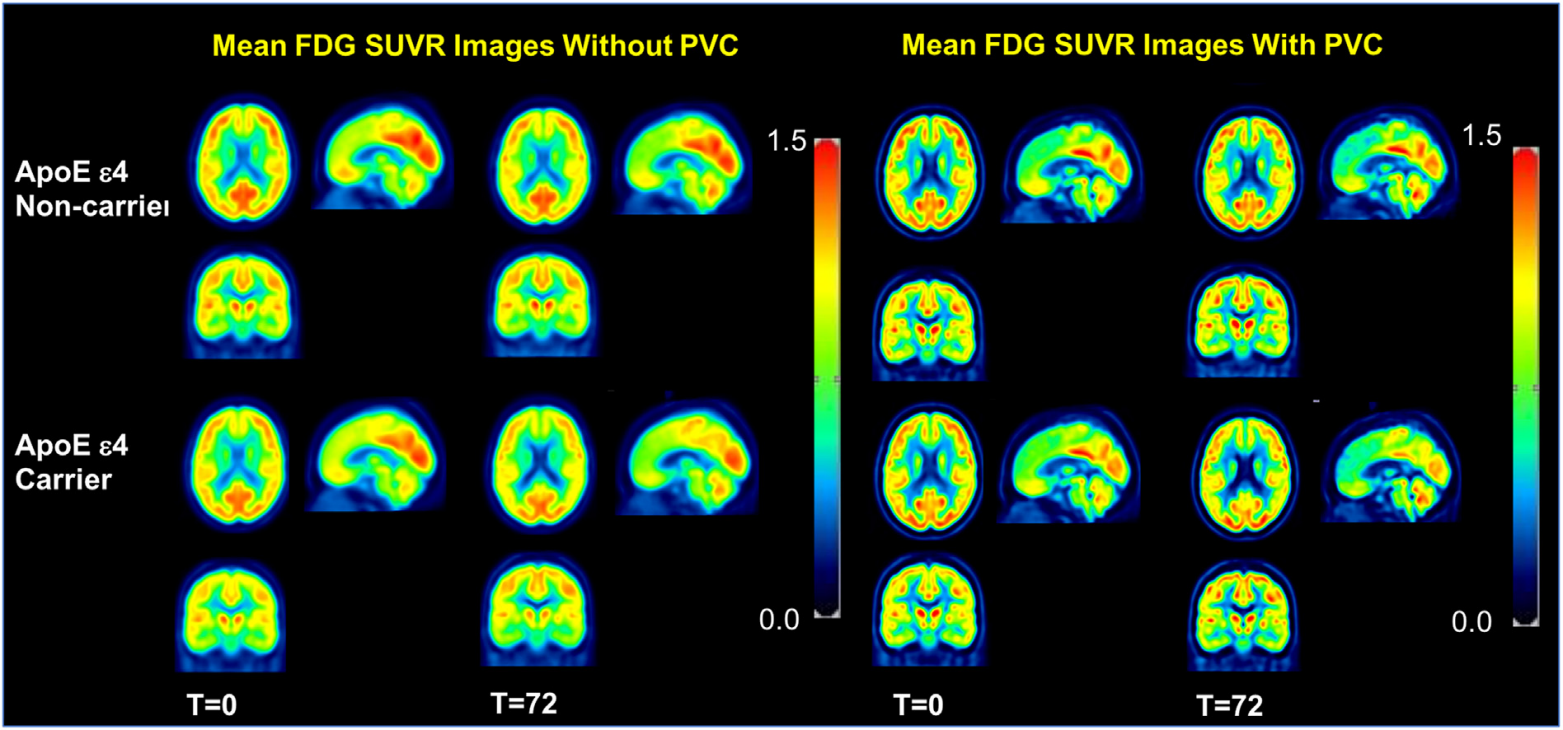
Mean SUVR Images with PVC and non-PVC. Mean images were generated by computing the mean of images from ApoE ε4 carriers and non-carriers separately at baseline and 72 months. These images have been corrected for partial volume effects. Note that the mean images are averaged over all participants in each group from non-PVC (left) and PVC (right) data.

Notably, in the non-PVC SUVR image-based SPM analysis, 14 clusters exhibit significantly decreased FDG uptake at 72 months compared to 0 months among ApoE ε4 carriers No significant clusters were detected among ApoE ε4 non-carriers. In order to create an anatomical visualization of the effects of PVC on spatial resolution and image contrast, we displayed one representative ApoE ε4 carrier and one ApoE ε4 non-carrier with and without PVC in Fig. 4. At the individual level, Fig. 4 clearly demonstrates an increase in spatial resolution and contrast after PVC. The results at the individual level are quite consistent when averaged over our studied population as illustrated in Fig. 5: mean FDG SUVR images at 0 months and 72 months averaged over all ApoE ε4 carriers and non-carriers.

We also compared baseline FDG-PET scans between Apoe ε4 carriers and non-carriers to understand basal differences in FDG. We found no significant ApoE-associated voxels in either PVC or non-PVC data (two-sample t-test, cluster level FWER <0.05; uncorrected voxel p < .001).

## 4 Discussion

In this study, we attempt to evaluate the spatial effects of the ApoE ɛ4 allele on metabolic changes in MCI measured by FDG PET using a PVC-based method to accurately assess longitudinal changes in FDG. In our sample of ApoE ɛ4 carriers and non-carriers with MCI, there was a clear longitudinal metabolic pattern in several brain regions that differed between ApoE ɛ4 carriers and ApoE ɛ4 non-carriers. Specifically, we found that the parietal cortex, temporal cortex, posterior cingulate, amygdala, caudate, and thalamic regions show greater longitudinal decreases in FDG tracer uptake in ApoE ε4 carriers compared to noncarriers in MCI patients. Importantly, these findings are unlikely to reflect differences in cognitive status, baseline age, education, follow-up interval and atrophy as these variables were controlled for in our analyses. Overall, by taking a longitudinal approach and correcting for partial volume effects, our study elucidates an ApoE ε4-related metabolic pattern in MCI patients.

The vast majority of previous studies linking the ApoE ε4 genotype to age-related decreases in cerebral glucose metabolism and blood flow have not been in patients with MCI (Breslow et al., 1982; Farrer et al.; Scarmeas et al., 2004; Thambisetty et al., 2010a; Wolk and Dickerson, 2010). For example, Thambisetty et al. found greater longitudinal decline in cerebral blood flow in non-demented ApoE ε4 carriers compared to non-carriers in the temporal, parietal, and frontal cortices (Thambisetty et al., 2010a). Consistent with a blood flow decrease of −6% to −2% in ApoE ε4 carriers compared to non-carriers in their study, we also observe similar regional decreases in FDG uptake in ε4 carriers compared to non-carriers in patients with MCI. Drzezga compared FDG uptake in ApoE ε4 carriers to non-carriers with Alzheimer's and found a pattern of cerebral hypometabolism in the parietal, temporal, and cingulate cortical areas in ApoE ε4 carriers compared to non-carriers (Drzezga et al., 2005). In the context of MCI, we find, in addition to the temporal cortex, parietal cortex, and posterior cingulate, previously unreported ApoE ε4-associated metabolic effects in the caudate, putamen, and amygdala. The ApoE ε4 allele has been previously shown to induce amygdalar atrophy in the context of MCI and AD but has been not reported to change metabolic status in either MCI or AD (Tang et al., 2015). Likewise, distinct patterns of striatal atrophy and increased Aβ deposition, but not metabolic change, have been observed in ApoE ε4 carriers compared to non-carriers in Alzheimer's disease (Cohen and Klunk, 2014; Pievani et al., 2013). By taking a longitudinal approach, we were able to elucidate previously unreported metabolic changes in the limbic region and striatum of ApoE ε4 carriers compared to non-carriers with MCI. A paired t-test assessing voxel-wise FDG at 72 months and 0 months complement the results of the more extensive longitudinal mixed-effects ROI-based modeling (Table 2, Table 3). Our findings, combined with existing data showing the increased power of incorporating ApoE ε4 carrier status in predicting conversion from MCI to AD, underscore the importance of studying the metabolic effects of the ApoE ε4 allele specifically among individuals with MCI.

While previous studies have explored ApoE ε4-dependent changes in subjects with AD and MCI, these studies have largely been cross-sectional (Drzezga et al., 2005; Hirono et al., 2002; Matsuda, 2001; Reiman et al., 1996; Roher et al., 2012; Scarmeas et al., 2004). Longitudinal studies are powerful in their ability to assess pathological changes over time rather than at a single time point. Differences in participant survival rates and changes in PET imaging measurement over time lead to variable FDG SUVR values. As a result, apparent ApoE ε4-mediated decreases in FDG SUVR at any given time point may not be statistically significant. Longitudinal studies overcome this challenge by considering patterns of tracer uptake over time. To our knowledge, our work represents the longest lasting longitudinal study on ApoE ε4 dependent changes in MCI.

To minimize partial volume effects in FDG SUVR measurements resulting from low image resolution in PET images from this multi-site collaboration, we applied PVC using previously published methods (Tohka and Reilhac, 2008). Poor image resolution can lead to blurring of images and subsequent spill-out or spill-in of tracer signal into surrounding regions determined by a point spread function. We applied a deconvolution based partial volume correction to adjust for these effects. Our ROI based results confirm that the PVC is necessary to elucidate ApoE ε4-mediated changes in FDG uptake in small regions. Specifically, we observe a near but not significant ApoE ε4-mediated effect in FDG uptake in the superior frontal cortex and amygdala (*p* = .065, 0.094 for superior frontal and amygdala, respectively) without PVC for patients with MCI. However, after correcting for the partial volume effect, we observed a significant ApoE ε4-mediated effect in these regions. Similarly, using a paired t-test voxelwise analysis, we observed fewer significant clusters in which FDG at 72 month <0 month in both the ApoE ε4 carrier group (23 clusters with PVC and 14 clusters without PVC) and ApoE ε4 non-carrier group (4 clusters with PVC and 0 clusters without PVC). Both the ROI-based and voxelwise findings underscore the importance of correcting for partial volume effects for accurate FDG measurement during longitudinal aging studies involving atrophy of brain regions that are small relative to the PET spatial resolution. Further, as a result of applying PVC, we are confident that changes in FDG reported here are genuine metabolic changes associated with ApoE ε4 rather than due to volume-related or technical biases.

As a limitation to our study, it should be noted that other pathologies, such as intraneuronal protein deposits and cerebrovascular disease, may explain differences in glucose hypometabolism between ApoE ε4 carriers and non-carriers in our study. For example, white matter hyperintensities, or areas of increased T2-MRI signal due to small vessel cerebrovascular disease, have previously been shown to contribute to glucose metabolism in AD (Prins and Scheltens, 2015; Tosto et al., 2015). Baseline differences in cerebrovascular disease between ApoE ε4 carriers and non-carriers in our cohort may partially explain differences in glucose hypometabolism. Likewise, neurofibrillary tangles, composed of intracellular deposits of tau protein, and extracellular beta-amyloid plaques can occur before the onset of cognitive symptoms and have been shown to promote glucose hypometabolism in AD (Beason-Held et al., 2013; Bischof et al., 2016; Ingelsson et al., 2004). Because only 56% of our study participants had baseline amyloid (CSF and amyloid imaging) data available, we were unable to control for baseline amyloid positivity. As a result, differences in the severity of amyloid beta and tau proteinopathy between ApoE ε4 carriers and non-carriers in our cohort may lead to observed differences in hypometabolism.

Due to limited sample size, a lack of sex and ApoE ε4 subtype ((ε4/ ε4), (ε4/ ε2), and (ε4/ ε3)) specific analysis is a further limitation to our study. Indeed, recent studies have found sex-specific differences in AD biomarkers and pathologic changes in MCI and AD (Holland et al., 2013; Hua et al., 2010; Lin et al., 2015; Skup et al., 2011; Sundermann et al., 2017). To avoid potential confounding due to sex, we included sex as a covariate in all of our analyses. Future studies may focus on analyzing sex-specific associations between the ApoE ε4 allele and FDG-PET in MCI. Human and transgenic mice experiments have consistently shown differences in AD risk and biomarker levels between subjects with ε4, ε3, and ε2 alleles (Liu et al., 2013). Notably, the ApoE ε2 allele has been previously shown to have a neuroprotective effect in Alzheimer's disease (Corder et al., 1994). As the ADNI cohort sample increases, we hope to investigate ApoE ε4 subtype-specific effects on longitudinal FDG uptake in MCI and AD patients in future studies.

It should also be noted that our cohort selection method used cognitive staging and not biomarker profiles. The new NIA-AA research framework regards Alzheimer's disease as a continuum with biomarker profile and cognitive staging as independent sources of information (Jack et al., 2018). To accurately identify where on this continuum our study participants are, biomarkers, such as Aβ 1–42, CSF tau, [^18^F]AV1451 PET, [^11^C]PIB PET, and [^18^F]AV45, must be profiled. A biomarker-based definition of AD helps us move away from defining AD as a clinical syndrome with or without evidence of neuropathologic change towards a biological disease marked by distinct biomarker changes. However, given that we are limited by the lack of available biomarkers in ADNI, our cohort should more accurately be described as participants with a clinical definition of MCI rather than a biological one.

## 5 Conclusion

We assessed the effect of the ApoE ε4 allele on glucose metabolism in MCI and elucidated a novel, regional pattern of glucose hypometabolism over up to 84-months longitudinal follow-up period (median = 72 months). Specifically, the superior fontal, parietal, lateral temporal, medial temporal, caudate, thalamus, and post-cingulate, and amygdala regions show greater longitudinal decreases in FDG uptake in ApoE ε4 carriers compared to non-carriers in the context of MCI. Partial volume correction improved quantitative FDG PET sensitivity in the study. Overall, our work represents one of the longest FDG studies of MCI, and uncovers a novel ApoE ε4-associated metabolic dynamic pattern in patients with MCI.

The following are the supplementary data related to this article.

**Supplementary Fig. 2:**
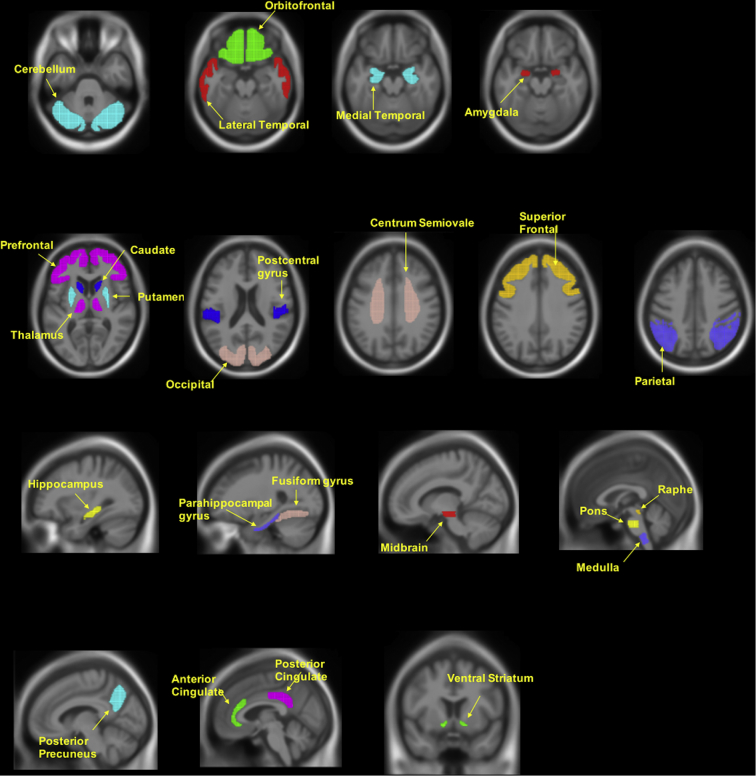
ROIs drawn superimposed on standard MNI152 MRI template. Representative ROIs are shown drawn on the MNI152 template using PMOD. ROI SUVRs were obtained by applying the above ROIs to SUVR images in the MNI space.

Supplementary Table 1 ADNI subjects included in the present study. Scans present and ApoE e4 carrier status are noted for each subject. Supplementary Table 1

## References

[bb0005] Beason-Held L.L., Goh J.O., An Y., Kraut M.A., O'Brien R.J., Ferrucci L., Resnick S.M. (2013). Changes in brain function occur years before the onset of cognitive impairment. J. Neurosci..

[bb0010] Bischof G.N., Jessen F., Fliessbach K., Dronse J., Hammes J., Neumaier B., Onur O., Fink G.R., Kukolja J., Drzezga A., van Eimeren T. (2016). Impact of tau and amyloid burden on glucose metabolism in Alzheimer's disease. Ann. Clin. Transl. Neurol..

[bb0015] Breslow J.L., Zannis V.I., SanGiacomo T.R., Third J.L., Tracy T., Glueck C.J. (1982). Studies of familial type III hyperlipoproteinemia using as a genetic marker the apoE phenotype E2/2. J. Lipid Res..

[bb0020] Bruscoli M., Lovestone S. (2004). Is MCI really just early dementia? A systematic review of conversion studies. Int. Psychogeriatrics..

[bb0025] Chan D., Janssen J.C., Whitwell J.L., Watt H.C., Jenkins R., Frost C., Rossor M.N., Fox N.C. (2003). Change in rates of cerebral atrophy over time in early-onset Alzheimer's disease: longitudinal MRI study. Lancet.

[bb0030] Chen X., Zhou Y., Wang R., Cao H., Reid S., Gao R., Han D. (2016). Potential clinical value of multiparametric pet in the prediction of Alzheimer's disease progression. PLoS One.

[bb0035] Chen Y., Denny K.G., Harvey D., Farias S.T., Mungas D., DeCarli C., Beckett L. (2017). Progression from normal cognition to mild cognitive impairment in a diverse clinic-based and community-based elderly cohort. Alzheimers Dement..

[bb0040] Cohen A.D., Klunk W.E. (2014). Early detection of Alzheimer's disease using PiB and FDG PET. Neurobiol. Dis..

[bb0045] Cope T.E., Rittman T., Borchert R.J., Jones P.S., Vatansever D., Allinson K., Passamonti L., Vazquez Rodriguez P., Bevan-Jones W.R., O'Brien J.T., Rowe J.B. (2018). Tau burden and the functional connectome in Alzheimer's disease and progressive supranuclear palsy. Brain.

[bb0050] Corder E.H., Saunders A.M., Risch N.J., Strittmatter W.J., Schmechel D.E., Gaskell P.C., Rimmler J.B., Locke P.A., Conneally P.M., Schmader K.E., Small G.W., Roses A.D., Haines J.L., Pericak-Vance M.A. (1994). Protective effect of apolipoprotein E type 2 allele for late onset Alzheimer disease. Nat. Genet..

[bb0055] Dickens A.M., Yoo S.W., Chin A.C., Xu J., Johnson T.P., Trout A.L., Hauser K.F., Haughey N.J. (2017). Chronic low-level expression of HIV-1 tat promotes a neurodegenerative phenotype with aging. Sci. Rep..

[bb0060] Drzezga A., Riemenschneider M., Strassner B., Grimmer T., Peller M., Knoll A., Wagenpfeil S., Minoshima S., Schwaiger M., Kurz A. (2005). Cerebral glucose metabolism in patients with AD and different APOE genotypes. Neurology.

[bb0065] Dukart J., Bertolino A. (2014). When structure affects function--the need for partial volume effect correction in functional and resting state magnetic resonance imaging studies. PLoS One.

[bb0070] Elias-Sonnenschein L.S., Viechtbauer W., Ramakers I.H.G.B., Verhey F.R.J., Visser P.J. (2011). Predictive value of APOE-ε4 allele for progression from MCI to AD-type dementia: a meta-analysis. J. Neurol. Neurosurg. Psychiatry.

[bb0075] Farrer L.A., Cupples L.A., Haines J.L., Hyman B., Kukull W.A., Mayeux R., Myers R.H., Pericak-Vance M.A., Risch N., van Duijn C.M. (1997). Effects of age, sex, and ethnicity on the association between Apolipoprotein E genotype and Alzheimer disease: a Meta-analysis. JAMA J. Am. Med. Assoc..

[bb0085] Feussner G., Feussner V., Hoffmann M.M., Lohrmann J., Wieland H., Mrz W. (1998). Molecular basis of type III hyperlipoproteinemia in Germany. Hum. Mutat..

[bb0095] Fouquet M., Desgranges B., Landeau B., Duchesnay E., Mezenge F., de la Sayette V., Viader F., Baron J.C., Eustache F., Chetelat G. (2009). Longitudinal brain metabolic changes from amnestic mild cognitive impairment to Alzheimer's disease. Brain.

[bb0100] Friedman G., Froom P., Sazbon L., Grinblatt I., Shochina M., Tsenter J., Babaey S., Yehuda A.B., Groswasser Z. (1999). Apolipoprotein E- 4 genotype predicts a poor outcome in survivors of traumatic brain injury. Neurology..

[bb0105] Gao R., Zhang G., Chen X., Yang A., Smith G., Wong D.F., Zhou Y. (2016). CSF biomarkers and its associations with18F-AV133 cerebral VMAT2 binding in Parkinson's disease-a preliminary report. PLoS One.

[bb0110] Genin E., Hannequin D., Wallon D., Sleegers K., Hiltunen M., Combarros O., Bullido M.J., Engelborghs S., De Deyn P., Berr C., Pasquier F., Dubois B., Tognoni G., Fiévet N., Brouwers N., Bettens K., Arosio B., Coto E., Del Zompo M., Mateo I., Epelbaum J., Frank-Garcia A., Helisalmi S., Porcellini E., Pilotto A., Forti P., Ferri R., Scarpini E., Siciliano G., Solfrizzi V., Sorbi S., Spalletta G., Valdivieso F., Vepsäläinen S., Alvarez V., Bosco P., Mancuso M., Panza F., Nacmias B., Boss P., Hanon O., Piccardi P., Annoni G., Seripa D., Galimberti D., Licastro F., Soininen H., Dartigues J.F., Kamboh M.I., Van Broeckhoven C., Lambert J.C., Amouyel P., Campion D. (2011). APOE and Alzheimer disease: a major gene with semi-dominant inheritance. Mol. Psychiatry.

[bb0115] Gottesman R.F., Schneider A.L.C., Zhou Y., Chen X., Green E., Gupta N., Knopman D.S., Mintz A., Rahmim A., Sharrett A.R., Wagenknecht L.E., Wong D.F., Mosley T.H. (2016). The ARIC-PET amyloid imaging study: brain amyloid differences by age, race, sex, and APOE. Neurology..

[bb0120] Grijalva-Eternod C.S., Wells J.C.K., Cortina-Borja M., Salse-Ubach N., Tondeur M.C., Dolan C., Meziani C., Wilkinson C., Spiegel P., Seal A.J. (2012). The double burden of obesity and malnutrition in a protracted emergency setting: a cross-sectional study of Western Sahara refugees. PLoS Med..

[bb0125] Hirono N., Hashimoto M., Yasuda M., Ishii K., Sakamoto S., Kazui H., Mori E. (2002). The effect of APOE epsilon4 allele on cerebral glucose metabolism in AD is a function of age at onset. Neurology.

[bb0130] Hixson J.E., Vernier D.T. (1990). Restriction isotyping of human apolipoprotein E by gene amplification and cleavage with HhaI. J. Lipid Res..

[bb0135] Holland D., Desikan R.S., Dale A.M., McEvoy L.K. (2013). Higher rates of decline for women and apolipoprotein e ε4 carriers. Am. J. Neuroradiol..

[bb0140] Hua X., Hibar D.P., Lee S., Toga A.W., Jack C.R., Weiner M.W., Thompson P.M. (2010). Sex and age differences in atrophic rates: An ADNI study with n=1368 MRI scans. Neurobiol. Aging.

[bb0145] Hughes C.P., Berg L., Danziger W.L. (1982). A new clinical scale for the staging of dementia. Br. J. Psychiatry.

[bb0150] Ingelsson M., Fukumoto H., Newell K.L., Growdon J.H., Hedley-Whyte E.T., Frosch M.P., Albert M.S., Hyman B.T., Irizarry M.C. (2004). Early Aβ accumulation and progressive synaptic loss, gliosis, and tangle formation in AD brain. Neurology..

[bb0155] Jack C.R., Knopman D.S., Jagust W.J., Petersen R.C., Weiner M.W., Aisen P.S., Shaw L.M., Vemuri P., Wiste H.J., Weigand S.D., Lesnick T.G., Pankratz V.S., Donohue M.C., Trojanowski J.Q. (2013). Update on hypothetical model of Alzheimer's disease biomarkers. Lancet Neurol..

[bb0160] Jack C.R., Bennett D.A., Blennow K., Carrillo M.C., Dunn B., Haeberlein S.B., Holtzman D.M., Jagust W., Jessen F., Karlawish J., Liu E., Molinuevo J.L., Montine T., Phelps C., Rankin K.P., Rowe C.C., Scheltens P., Siemers E., Snyder H.M., Sperling R., Elliott C., Masliah E., Ryan L., Silverberg N. (2018). NIA-AA research framework: toward a biological definition of Alzheimer's disease. Alzheimers Dement..

[bb0165] Jahn H. (2013). Memory loss in alzheimer's disease. Dialogues Clin. Neurosci..

[bb0175] Jonasson L.S., Axelsson J., Riklund K., Boraxbekk C.J. (2017). Simulating effects of brain atrophy in longitudinal PET imaging with an anthropomorphic brain phantom. Phys. Med. Biol..

[bb0180] Kadotani H., Kadotani T., Young T., Peppard P.E., Finn L., Colrain I.M., Murphy G.M., Mignot E. (2001). Association between apolipoprotein E epsilon4 and sleep-disordered breathing in adults. JAMA.

[bb0185] Kaufer D.I., Cummings J.L., Ketchel P., Smith V., MacMillan A., Shelley T., Lopez O.L., DeKosky S.T. (2000). Validation of the NPI-Q, a brief clinical form of the neuropsychiatric inventory. J. Neuropsychiatry Clin. Neurosci..

[bb0190] Knopman D.S., Jack C.R., Wiste H.J., Lundt E.S., Weigand S.D., Vemuri P., Lowe V.J., Kantarci K., Gunter J.L., Senjem M.L., Mielke M.M., Roberts R.O., Boeve B.F., Petersen R.C. (2014). 18F-fluorodeoxyglucose positron emission tomography, aging, and apolipoprotein E genotype in cognitively normal persons. Neurobiol. Aging.

[bb0195] Lehtovirta M., Kuikka J., Helisalmi S., Hartikainen P., Mannermaa A., Ryynänen M., PSr Riekkinen, Soininen H. (1998). Longitudinal SPECT study in Alzheimer's disease: relation to apolipoprotein E polymorphism. J. Neurol. Neurosurg. Psychiatry.

[bb0200] Lin K.A., Choudhury K.R., Rathakrishnan B.G., Marks D.M., Petrella J.R., Doraiswamy P.M. (2015). Marked gender differences in progression of mild cognitive impairment over 8 years. Alzheimer's Dement. Transl. Res. Clin. Interv..

[bb0205] Liu C.-C., Liu C.-C., Kanekiyo T., Xu H., Bu G. (2013). Apolipoprotein E and Alzheimer disease: risk, mechanisms and therapy. Nat. Rev. Neurol..

[bb0210] Matsuda H. (2001). Cerebral blood flow and metabolic abnormalities in Alzheimer's disease. Ann. Nucl. Med..

[bb0215] Mecca A.P., Barcelos N.M., Wang S., Brück A., Nabulsi N., Planeta-Wilson B., Nadelmann J., Benincasa A.L., Ropchan J., Huang Y., Gelernter J., Van Ness P.H., Carson R.E., van Dyck C.H. (2018). Cortical β-amyloid burden, gray matter, and memory in adults at varying APOE ε4 risk for Alzheimer's disease. Neurobiol. Aging.

[bb0220] Misra C., Fan Y., Davatzikos C. (2009). Baseline and longitudinal patterns of brain atrophy in MCI patients, and their use in prediction of short-term conversion to AD: Results from ADNI. Neuroimage.

[bb0225] Moreno-Grau S., Rodríguez-Gómez O., Sanabria Á., Pérez-Cordón A., Sánchez-Ruiz D., Abdelnour C., Valero S., Hernández I., Rosende-Roca M., Mauleón A., Vargas L., Lafuente A., Gil S., Santos-Santos M.Á., Alegret M., Espinosa A., Ortega G., Guitart M., Gailhajanet A., de Rojas I., Sotolongo-Grau Ó., Ruiz S., Aguilera N., Papasey J., Martín E., Peleja E., Lomeña F., Campos F., Vivas A., Gómez-Chiari M., Tejero M.Á., Giménez J., Serrano-Ríos M., Orellana A., Tárraga L., Ruiz A., Boada M. (2017). Exploring APOE genotype effects on Alzheimer's disease risk and amyloid β burden in individuals with subjective cognitive decline: the FundacioACE healthy brain initiative (FACEHBI) study baseline results. Alzheimers Dement..

[bb0230] Mosconi L., Perani D., Sorbi S., Herholz K., Nacmias B., Holthoff V., Salmon E., Baron J.C., De Cristofaro M.T.R., Padovani A., Borroni B., Franceschi M., Bracco L., Pupi A. (2004). MCI conversion to dementia and the APOE genotype: a prediction study with FDG-PET. Neurology.

[bb0235] Mosconi L., Mistur R., Switalski R., Tsui W.H., Glodzik L., Li Y., Pirraglia E., De Santi S., Reisberg B., Wisniewski T., De Leon M.J. (2009). FDG-PET changes in brain glucose metabolism from normal cognition to pathologically verified Alzheimer's disease. Eur. J. Nucl. Med. Mol. Imaging.

[bb0240] Mosconi L., Berti V., Glodzik L., Pupi A., De Santi S., De Leon M.J. (2010). Pre-clinical detection of Alzheimer's disease using FDG-PET, with or without amyloid imaging. J. Alzheimers Dis..

[bb0245] Niethammer M., Feigin A., Eidelberg D. (2012). Functional neuroimaging in Parkinson's disease. Cold Spring Harb. Perspect. Med..

[bb0250] Pangman V.C., Sloan J., Guse L. (2000). An examination of psychometric properties of the mini-mental state examination and the standardized mini-mental state examination: implications for clinical practice. Appl. Nurs. Res..

[bb0260] Petersen R.C., Doody R., Kurz A., Mohs R.C., Morris J.C., Rabins P.V., Ritchie K., Rossor M., Thal L., Winblad B. (2001). Current concepts in mild cognitive impairment. Arch. Neurol..

[bb0265] Petersen R.C., Thomas R.G., Grundman M., Bennett D., Doody R., Ferris S., Galasko D., Jin S., Kaye J., Levey A., Pfeiffer E., Sano M., van Dyck C.H., Thal L.J., Alzheimer's Disease Cooperative Study Group (2005). Vitamin E and donepezil for the treatment of mild cognitive impairment. N. Engl. J. Med..

[bb0270] Pfeffer R.I., Kurosaki T.T., Harrah C.H., Chance J.M., Filos S. (1982). Measurement of functional activities in older adults in the community. J. Gerontol..

[bb0275] Pievani M., Bocchetta M., Boccardi M., Cavedo E., Bonetti M., Thompson P.M., Frisoni G.B. (2013). Striatal morphology in early-onset and late-onset Alzheimer's disease: a preliminary study. Neurobiol. Aging.

[bb0280] Prins N.D., Scheltens P. (2015). White matter hyperintensities, cognitive impairment and dementia: An update. Nat. Rev. Neurol..

[bb0285] Rajan K.B., Wilson R.S., Weuve J., Barnes L.L., Evans D. a (2015). Cognitive impairment 18 years before clinical diagnosis of Alzheimer disease dementia. Neurology.

[bb0290] Reiman E.M., Caselli R.J., Yun L.S., Chen K.W., Bandy D., Minoshima S., Thibodeau S.N., Osborne D. (1996). Preclinical evidence of Alzheimers disease in persons homozygous for the ε-4 allele for Apolipoprotein E. N. Engl. J. Med..

[bb0295] Resnick S.M., Sojkova J., Zhou Y., An Y., Ye W., Holt D.P., Dannals R.F., Mathis C.A., Klunk W.E., Ferrucci L., Kraut M.A., Wong D.F. (2010). Longitudinal cognitive decline is associated with fibrillar amyloid-beta measured by [11C]PiB. Neurology..

[bb0305] Roher A.E., Debbins J.P., Malek-Ahmadi M., Chen K., Pipe J.G., Maze S., Belden C., Maarouf C.L., Thiyyagura P., Mo H., Hunter J.M., Kokjohn T.A., Walker D.G., Kruchowsky J.C., Belohlavek M., Sabbagh M.N., Beach T.G. (2012). Cerebral blood flow in Alzheimer’s disease. Vasc. Health Risk Manag..

[bb0310] Rosén C., Hansson O., Blennow K., Zetterberg H., Rosen C., Hansson O., Blennow K., Zetterberg H. (2013). Fluid biomarkers in Alzheimer's disease - current concepts. Mol. Neurodegener..

[bb0320] Scarabino D., Broggio E., Gambina G., Maida C., Gaudio M.R., Corbo R.M. (2016). Apolipoprotein E genotypes and plasma levels in mild cognitive impairment conversion to Alzheimer's disease: a follow-up study. Am. J. Med. Genet. Part B Neuropsychiatr. Genet..

[bb0325] Scarmeas N., Anderson K.E., Hilton J., Park A., Habeck C., Flynn J., Tycko B., Stern Y. (2004). APOE-dependent PET patterns of brain activation in Alzheimer disease. Neurology.

[bb0330] Schmidt S., Barcellos L.F., DeSombre K., Rimmler J.B., Lincoln R.R., Bucher P., Saunders A.M., Lai E., Martin E.R., Vance J.M., Oksenberg J.R., Hauser S.L., Pericak-Vance M. a, Haines J.L. (2002). Association of polymorphisms in the apolipoprotein E region with susceptibility to and progression of multiple sclerosis. Am. J. Hum. Genet..

[bb0335] Skup M., Zhu H., Wang Y., Giovanello K.S., Lin J. an, Shen D., Shi F., Gao W., Lin W., Fan Y., Zhang H. (2011). Sex differences in grey matter atrophy patterns among AD and aMCI patients: Results from ADNI. Neuroimage..

[bb0340] Small G.W., Ercoli L.M., Silverman D.H., Huang S.C., Komo S., Bookheimer S.Y., Lavretsky H., Miller K., Siddarth P., Rasgon N.L., Mazziotta J.C., Saxena S., Wu H.M., Mega M.S., Cummings J.L., Saunders A.M., Pericak-Vance M.A., Roses A.D., Barrio J.R., Phelps M.E. (2000). Cerebral metabolic and cognitive decline in persons at genetic risk for Alzheimer's disease. Proc. Natl. Acad. Sci. U. S. A..

[bb0345] Sundermann E.E., Biegon A., Rubin L.H., Lipton R.B., Landau S., Maki P.M. (2017). Does the female advantage in verbal memory contribute to underestimating Alzheimer's disease pathology in women versus men?. J. Alzheimers Dis..

[bb0350] Tang X., Hollandb D., Dale A.M., Miller M.I. (2015). APOE affects the volume and shape of the amygdala and the Hippocampus in mild cognitive impairment and Alzheimer's disease: age matters. J. Alzheimers Dis..

[bb0355] Thambisetty M., Beason-Held L., An Y., Kraut M.A., Resnick S.M. (2010). APOE epsilon4 genotype and longitudinal changes in cerebral blood flow in normal aging. Arch. Neurol..

[bb0360] Thambisetty M., Tripaldi R., Riddoch-Contreras J., Hye A., An Y., Campbell J., Sojkova J., Kinsey A., Lynham S., Zhou Y., Ferrucci L., Wong D.F., Lovestone S., Resnick S.M. (2010). Proteome-based plasma markers of brain amyloid-β deposition in non-demented older individuals. J. Alzheimers Dis..

[bb0365] Tohka J., Reilhac A. (2008). Deconvolution-based partial volume correction in Raclopride-PET and Monte Carlo comparison to MR-based method. Neuroimage.

[bb0370] Tosto G., Zimmerman M.E., Hamilton J.L., Carmichael O.T., Brickman A.M. (2015). The effect of white matter hyperintensities on neurodegeneration in mild cognitive impairment. Alzheimers Dement..

[bb0375] Winblad B., Palmer K., Kivipelto M., Jelic V., Fratiglioni L., Wahlund L.O., Nordberg A., Bäckman L., Albert M., Almkvist O., Arai H., Basun H., Blennow K., de Leon M., DeCarli C., Erkinjuntti T., Giacobini E., Graff C., Hardy J., Jack C., Jorm A., Ritchie K., van Duijn C., Visser P., Petersen R.C. (2004). Mild cognitive impairment--beyond controversies, towards a consensus: report of the international working group on mild cognitive impairment. J. Intern. Med..

[bb0380] Wolk D. a, Dickerson B.C. (2010). Apolipoprotein E (APOE) genotype has dissociable effects on memory and attentional-executive network function in Alzheimer's disease. Proc. Natl. Acad. Sci. U. S. A..

[bb0385] Zhang Z., Mu J., Li J., Li W., Song J. (2013). Aberrant Apolipoprotein E expression and cognitive dysfunction in patients with Poststroke depression. Genet. Test. Mol. Biomarkers.

[bb0390] Zhou Y., Resnick S.M., Ye W., Fan H., Holt D.P., Klunk W.E., Mathis C.A., Dannals R., Wong D.F. (2007). Using a reference tissue model with spatial constraint to quantify [11C]Pittsburgh compound B PET for early diagnosis of Alzheimer's disease. Neuroimage..

