# Supplementary material for: The effect of ApoE ε4 on longitudinal brain region-specific glucose metabolism in patients with mild cognitive impairment: a FDG-PET study
Source: Neuroimage Clin. 2019 Mar 28;22:101795. doi: 10.1016/j.nicl.2019.101795 (PMC6449776; doi:10.1016/j.nicl.2019.101795)
Supplement: Supplementary Table 1 — ADNI subjects included in the present study. Scans present and ApoE e4 carrier status are noted for each subject. [file mmc1.docx]

| **Subject ID** | **ApoE e4 Status** | **Scan Present (months; 0=not present, 1=present)** | | | | | | | | | |
| --- | --- | --- | --- | --- | --- | --- | --- | --- | --- | --- | --- |
|  |  | 0 | 6 | 12 | 18 | 24 | 36 | 48 | 60 | 72 | 84 |
| 003_S_1074 | Non-carrier | 1 | 1 | 1 | 1 | 1 | 1 | 1 | 0 | 1 | 0 |
| 003_S_1122 | Non-carrier | 1 | 1 | 1 | 1 | 1 | 1 | 1 | 0 | 1 | 1 |
| 005_S_0546 | Carrier | 1 | 1 | 1 | 1 | 1 | 1 | 1 | 0 | 0 | 0 |
| 006_S_1130 | Carrier | 1 | 1 | 1 | 1 | 1 | 1 | 1 | 0 | 1 | 0 |
| 007_S_0101 | Carrier | 1 | 1 | 1 | 1 | 1 | 1 | 0 | 0 | 1 | 0 |
| 009_S_1030 | Non-carrier | 1 | 1 | 1 | 1 | 0 | 1 | 1 | 0 | 1 | 0 |
| 013_S_1186 | Non-carrier | 1 | 1 | 1 | 1 | 1 | 0 | 1 | 0 | 1 | 0 |
| 018_S_0142 | Carrier | 1 | 1 | 0 | 1 | 1 | 1 | 0 | 1 | 0 | 0 |
| 021_S_0626 | Non-carrier | 1 | 1 | 1 | 1 | 1 | 1 | 1 | 0 | 0 | 0 |
| 027_S_0408 | Non-carrier | 1 | 1 | 1 | 1 | 1 | 1 | 0 | 1 | 0 | 1 |
| 029_S_1318 | Non-carrier | 1 | 1 | 1 | 1 | 1 | 1 | 1 | 0 | 1 | 0 |
| 031_S_0294 | Non-carrier | 1 | 1 | 1 | 1 | 1 | 1 | 0 | 1 | 0 | 0 |
| 032_S_0214 | Carrier | 1 | 1 | 1 | 1 | 1 | 1 | 0 | 1 | 0 | 1 |
| 033_S_0906 | Carrier | 1 | 1 | 1 | 1 | 1 | 1 | 1 | 0 | 1 | 0 |
| 036_S_0945 | Non-carrier | 1 | 1 | 1 | 1 | 1 | 1 | 1 | 0 | 0 | 0 |
| 037_S_0150 | Non-carrier | 1 | 1 | 1 | 1 | 1 | 1 | 0 | 1 | 0 | 0 |
| 037_S_0377 | Carrier | 1 | 1 | 1 | 1 | 1 | 1 | 1 | 0 | 0 | 0 |
| 037_S_0552 | Carrier | 1 | 1 | 1 | 1 | 1 | 0 | 0 | 1 | 0 | 0 |
| 037_S_0566 | Carrier | 1 | 1 | 1 | 1 | 1 | 0 | 0 | 1 | 0 | 0 |
| 037_S_1078 | Carrier | 1 | 1 | 1 | 1 | 1 | 1 | 1 | 1 | 0 | 1 |
| 041_S_0679 | Non-carrier | 1 | 1 | 1 | 1 | 1 | 1 | 0 | 1 | 0 | 1 |
| 041_S_1418 | Non-carrier | 1 | 1 | 1 | 1 | 1 | 1 | 1 | 0 | 1 | 0 |
| 052_S_1346 | Carrier | 1 | 1 | 1 | 1 | 1 | 1 | 1 | 0 | 1 | 0 |
| 053_S_0919 | Carrier | 1 | 1 | 1 | 1 | 1 | 1 | 0 | 1 | 0 | 1 |
| 057_S_1007 | Non-carrier | 1 | 1 | 1 | 1 | 1 | 1 | 0 | 1 | 0 | 0 |
| 073_S_0746 | Non-carrier | 1 | 1 | 1 | 1 | 1 | 1 | 0 | 1 | 0 | 1 |
| 099_S_0051 | Carrier | 1 | 1 | 1 | 1 | 1 | 1 | 0 | 0 | 1 | 0 |
| 099_S_0291 | Carrier | 1 | 1 | 1 | 1 | 1 | 1 | 0 | 0 | 1 | 0 |
| 114_S_0378 | Carrier | 1 | 1 | 1 | 1 | 1 | 1 | 0 | 0 | 1 | 1 |
| 114_S_1106 | Non-carrier | 1 | 1 | 1 | 1 | 1 | 1 | 0 | 1 | 0 | 1 |
| 114_S_1118 | Non-carrier | 1 | 1 | 1 | 1 | 1 | 1 | 0 | 1 | 0 | 1 |
| 116_S_0361 | Carrier | 1 | 1 | 1 | 1 | 1 | 1 | 0 | 1 | 0 | 1 |
| 126_S_0709 | Carrier | 1 | 1 | 1 | 1 | 0 | 1 | 0 | 1 | 0 | 1 |
| 127_S_0112 | Carrier | 1 | 1 | 1 | 1 | 1 | 1 | 0 | 1 | 0 | 1 |
| 127_S_0925 | Non-carrier | 1 | 1 | 1 | 1 | 1 | 1 | 1 | 0 | 1 | 0 |
| 127_S_1427 | Non-carrier | 1 | 1 | 1 | 1 | 1 | 1 | 0 | 1 | 0 | 0 |
| 128_S_0135 | Non-carrier | 1 | 1 | 1 | 1 | 1 | 1 | 1 | 0 | 1 | 0 |
| 128_S_0200 | Non-carrier | 1 | 1 | 1 | 1 | 1 | 1 | 1 | 0 | 1 | 0 |
| 128_S_0225 | Non-carrier | 1 | 1 | 1 | 1 | 1 | 1 | 1 | 0 | 1 | 0 |
| 128_S_0227 | Carrier | 1 | 0 | 1 | 1 | 1 | 1 | 1 | 1 | 0 | 1 |
| 128_S_1043 | Non-carrier | 1 | 1 | 1 | 1 | 0 | 1 | 0 | 1 | 0 | 0 |
| 129_S_1246 | Carrier | 1 | 1 | 1 | 1 | 1 | 1 | 1 | 0 | 1 | 0 |
| 130_S_0285 | Carrier | 1 | 1 | 1 | 1 | 1 | 1 | 0 | 1 | 0 | 1 |
| 130_S_0289 | Non-carrier | 1 | 1 | 1 | 1 | 1 | 1 | 0 | 1 | 0 | 0 |
| 137_S_0722 | Non-carrier | 1 | 1 | 1 | 1 | 1 | 1 | 1 | 0 | 1 | 0 |
| 137_S_0800 | Carrier | 1 | 1 | 1 | 1 | 1 | 1 | 1 | 0 | 1 | 0 |
| 137_S_0994 | Carrier | 1 | 1 | 1 | 1 | 1 | 1 | 1 | 0 | 1 | 0 |
| 137_S_1414 | Carrier | 1 | 1 | 1 | 1 | 1 | 1 | 0 | 1 | 0 | 0 |

**Supplementary Table 1: ADNI subjects included in the present study. Scans present and ApoE e4 carrier status are noted for each subject**
